# Ultrasound-guided microwave ablation versus surgery for low-risk solitary papillary thyroid microcarcinoma: a propensity-matched cohort study

**DOI:** 10.1530/EC-24-0366

**Published:** 2025-01-06

**Authors:** Yujie Ren, Yujiang Li, Xiaoqiu Chu, Guofang Chen, Xue Han, Yueting Zhao, Chao Liu, Jianhua Wang, Shuhang Xu

**Affiliations:** ^1^ Department of Endocrinology and Metabolism, Affiliated Hospital of Integrated Traditional Chinese and Western Medicine, Nanjing University of Chinese Medicine, Nanjing, China; ^2^ Key Laboratory of TCM Syndrome and Treatment of Yingbing (Thyroid Disease) of State Administration of Traditional Chinese Medicine, Jiangsu Province Academy of Traditional Chinese Medicine, Nanjing, China; ^3^ Department of General Surgery, Affiliated Hospital of Integrated Traditional Chinese and Western Medicine, Nanjing University of Chinese Medicine, Nanjing, China

**Keywords:** microwave ablation, surgery, recurrence, papillary thyroid microcarcinoma, propensity score matching

## Abstract

**Objective:**

To evaluate the therapeutic effects of microwave ablation (MWA) versus surgery in treating low-risk papillary thyroid microcarcinoma (PTMC) and to assess recurrence-free survival (RFS) in patients with and without the BRAFV600E mutation.

**Methods:**

Between August 2016 and September 2022, 158 patients diagnosed with low-risk PTMC treated with MWA and 288 patients who underwent surgical treatment were retrospectively analyzed. All patients were followed-up for over a year. Local tumor progression (LTP), RFS and adverse events associated with both treatments were monitored. Following propensity score matching (PSM), comparisons were made regarding LTP, RFS, complications and treatment variables.

**Results:**

Prior to matching, MWA patients were younger than those in the surgery group (38 (30.75, 47) vs 43 (34, 50.75), *P* = 0.000). Tumors treated with MWA had smaller maximum diameters (5.7 (4.6, 7.0) vs 6.9 (5.8, 8.6), *P* = 0.000) and volumes (70.7 (35.2, 120.9) vs 122.0 (63.9, 228.8), *P* = 0.000). After 1:1 PSM, each group contained 141 patients with comparable baseline characteristics. During the follow-up, LTP developed in nine patients: six in the MWA group and three in the surgery group. There were no cases of distant metastasis or cancer-related deaths. Adjusting for age, sex, tumor location and largest diameter, there was no significant association between treatment modality and recurrence (HR = 3.75, 95% CI: 0.94–14.98, *P* = 0.062). There were no significant differences in RFS between patients with and without the BRAFV600E mutation in both groups (*P* = 0.45 and 0.74, respectively). Furthermore, the incidence of complications was comparable between treatments.

**Conclusion:**

Both MWA and surgical treatment offer similar efficacy and safety profiles for managing low-risk PTMC. MWA may represent a viable alternative to conventional surgical approaches, especially for patients harboring the BRAFV600E mutation.

## Introduction

With advancements in high-resolution ultrasound (US) technology, the detection rate of thyroid cancer has risen (1). Papillary thyroid carcinoma (PTC), particularly papillary thyroid microcarcinoma (PTMC) with a maximum diameter of ≤1 cm, is the most prevalent type. Low-risk PTMC is characterized by a cytological diagnosis of PTC, the absence of clinical lymph node metastasis (LNM) or distant metastasis, no extrathyroidal extension (ETE) and the lack of invasion into the recurrent laryngeal nerve (RLN) or trachea, as confirmed by cytopathological examination (2). Currently, thyroid lobectomy (TL) remains the primary treatment for PTMC (3). While surgery effectively removes the primary tumor and reduces recurrence and LNM risks, it also entails complications such as the need for permanent medication and potential scarring. Given the indolent nature of most PTMCs, which generally have a favorable prognosis, concerns about overdiagnosis and overtreatment are growing. Active surveillance (AS) is now widely endorsed for low-risk PTMC because of its effectiveness and safety, offering a reliable alternative to surgery, which still carries risks of tumor growth and LNM (4).

As another treatment method, US-guided thermal ablation (TA) represents a viable option for low-risk PTMC. Current US-guided TA technologies primarily encompass laser ablation, radiofrequency ablation (RFA) and microwave ablation (MWA) (5). The long-term efficacy of TA compared with surgical treatments for low-risk PTMC warrants consideration. Meta-analyses by Shen *et al.* (6) and Cheng *et al.* (7) have compared the effectiveness and safety of TA and surgical treatments, revealing significantly lower complications, procedure times, hospitalization durations and costs in the TA group, with no marked differences in recurrence and metastasis rates. A study by Yan *et al.* (8) using propensity score matching (PSM) to compare surgical and RFA treatments in 884 PTMC patients found no significant association with recurrence. However, these studies are predominantly from limited research centers, and further validation is needed for comparisons between MWA and surgery.

The correlation between the BRAFV600E mutation and the clinical–pathological progression of PTMC remains contentious. A retrospective study by Lin *et al.* (9) on 60 single-focus PTMC patients with the BRAFV600E mutation indicated no occurrences of local tumor progression (LTP) or central LNM (CLNM), suggesting that RFA is an effective and safe treatment for single-focus PTMC with this mutation. The effectiveness and safety of MWA in treating low-risk PTMC with the BRAFV600E mutation still require further investigation.

The objectives of this study are to compare the therapeutic effects of MWA and the surgical treatment of low-risk PTMC and to evaluate the long-term efficacy and safety of MWA for treating PTMC with the BRAFV600E mutation.

## Materials and methods

### Subjects

This retrospective study was approved by the Institutional Review Board of the Affiliated Hospital of Integrated Traditional Chinese and Western Medicine (2023-LWKYZ-070). All patients received treatment at the same institution and provided written informed consent prior to any procedure.

Inclusion criteria are (i) diagnosis of PTC with a single focus, confirmed via thyroid fine-needle aspiration cytology (FNAC) or core needle biopsy (CNB) before treatment; (ii) maximum tumor diameter ≤10 mm; (iii) aged >16 years; (iv) no clinical or imaging evidence of capsular or extrathyroidal invasion, RLN or trachea invasion and lymph node or distant metastasis; and (v) a follow-up period of ≥12 months. Exclusion criteria are (i) the presence of other thyroid cancer types; (ii) PTMC with multiple foci; (iii) allergy to local anesthetics, painkillers and hemostatics used in the study; (iv) coagulation disorders (prothrombin time >18 s, prothrombin activity <40%); (v) pregnancy or lactation; and (vi) unavailability of clinical data.

**Figure 1:**
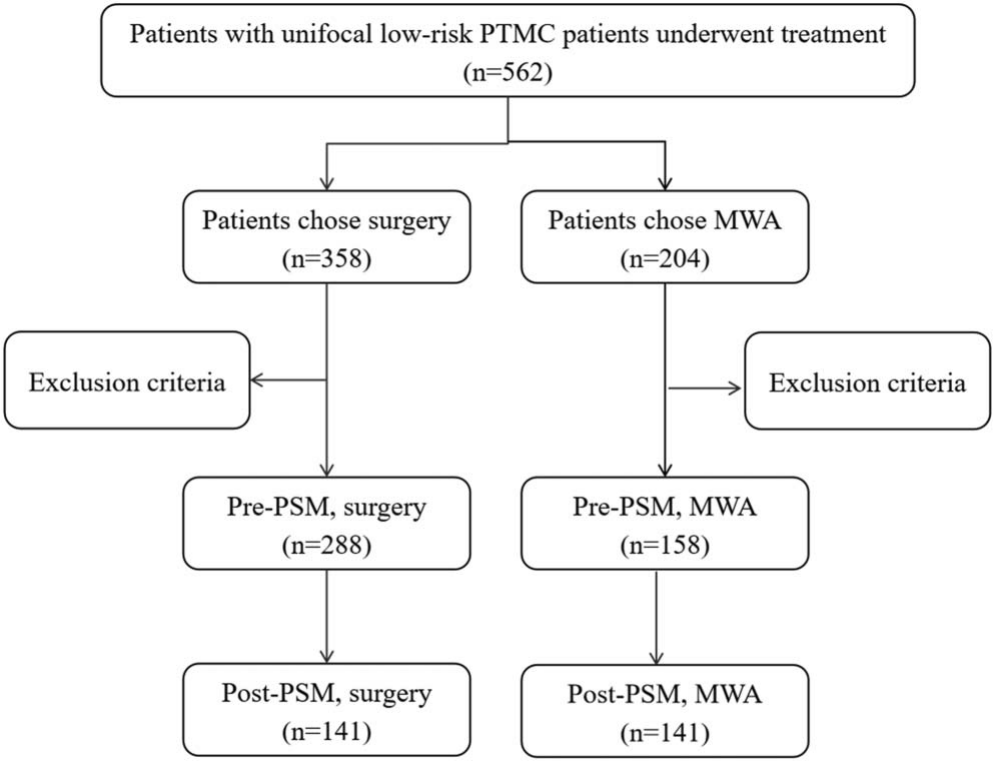
Flowchart of the study cohort.

From August 2016 to September 2022, 204 patients with malignant thyroid nodules who underwent MWA were initially considered. Of these, eight patients with multifocal tumors and 13 with PTC were excluded, and 38 were excluded because of follow-up periods of less than one year. Ultimately, 158 patients were included. In the surgery group, 358 patients with low-risk PTMC underwent surgery during the same period; 70 of these patients were followed up for less than a year. Finally, 288 patients who underwent surgery were included (Fig. 1).

### Pretreatment evaluation

All patients underwent comprehensive pretreatment evaluations including complete blood counts, thyroid function tests, coagulation profiles and imaging assessments such as US, chest CT and enhanced CT. These evaluations aimed to rule out ETE or any local or distant metastases. The tumor volume was calculated using the following formula: *V* = π*abc*/6, where *V* is the volume, *a* is the largest diameter measured on US and *b* and *c* are the other two perpendicular diameters.

### Treatment and follow-up

Thyroidectomy was conducted by high-volume surgeons with over 20 years of experience in thyroid surgery. The minimum surgical extent for each patient included TL and central lymph node dissection. If a nodule was preoperatively identified in the contralateral lobe, the surgeons might perform a partial or subtotal lobectomy of the contralateral gland or opt not to treat it based on the preoperative assessment and patient consultation. All excised suspicious lesions were analyzed via a frozen section. If malignancy was indicated in the contralateral lobe nodes, contralateral lobectomy was performed.

US-guided MWA was administered by an experienced physician using an MWA system (KY-2000, Canyon Medical, China) as previously reported (10). This system included a generator, a power distribution system and a cooled-shaft antenna measuring 1.6 mm in diameter and 10 cm in length, with a 3 mm gap from the electrode to the needle tip. The MWA system, operating at 2450 MHz and 35 W, induced coagulative necrosis in the thyroid tumors and surrounding tissues, which were ablated until they appeared hyperechoic. The residual heat from the antenna coagulated the puncture path to prevent tumor cell proliferation and implantation. During the procedure, intermittent communication with the patient aimed to minimize potential damage to the RLN. Contrast-enhanced US was performed immediately after MWA to assess the ablation area (11).

During follow-up, patients underwent thyroid function tests, US elastography and clinical evaluations. In the surgery group, follow-ups occurred every 6–12 months. All patients were advised to take levothyroxine post-treatment to maintain thyrotropin levels between 0.5 and 2 mU/L. In the MWA group, follow-ups were scheduled at 1, 3, 6 and 12 months and then every 6–12 months. Thyroid hormone suppression therapy was not prescribed post-MWA.

### End points and definitions

Primary outcome measures included LTP, distant metastasis, recurrence-free survival (RFS) and salvage surgery. LTP was defined as follows: (a) a persistently detected lesion at the ablated site confirmed by biopsy; (b) new recurrent PTMC separated from the treated tumor, confirmed by biopsy; and (c) cervical LNM confirmed by biopsy. Distant metastasis was identified through CT, positron emission tomography or bone scan in the presence of suspicious symptoms. RFS was calculated from the initiation of treatment to tumor recurrence or the last follow-up date. Salvage surgery was defined as surgery performed in the MWA group because of tumor progression or patient anxiety during follow-up.

Secondary outcome measures included treatment variables such as hospitalization, procedure time, estimated blood loss and cost. Procedure time encompassed the period from incision to closure, excluding anesthesia time. MWA costs covered the preoperative examination, the procedure itself, local anesthesia and microwave needle fees. Surgery costs included the preoperative examination, the surgical procedure, general anesthesia and the associated hospitalization expenses (hospital bed, nursing and postoperative medication fees).

Major and minor complications were recorded. Major complications primarily included RLN injury and hypoparathyroidism, while minor complications consisted of postoperative pain, bleeding, infection and others. RLN injury was defined as the impaired movement of one or both vocal cords on laryngoscopy, and permanent RLN injury was defined as an injury that did not resolve within 6 months. Hypoparathyroidism was characterized by a parathyroid hormone level <15 pg/mL after treatment, with distinctions made between temporary and permanent hypoparathyroidism based on recovery within 6 months.

### Statistical analysis

Continuously distributed variables conforming to a normal distribution are presented as mean ± standard deviation and were compared using the independent-sample *t*-test. Variables not normally distributed are depicted as medians with interquartile ranges and were analyzed using the Mann–Whitney U test. Categorical variables were presented as counts and percentages and compared using the chi-square or Fisher’s exact test. To control for inherent potential biases, 1:1 PSM was implemented across the two groups, considering age, sex, largest diameter, volume and location. This matching employed the nearest-neighbor method with a caliper distance of 0.05 and was conducted without replacement. Comparisons of patient characteristics and outcomes were made pre- and post-matching. RFS rates were computed using the Kaplan–Meier method and compared using the log-rank test. Both univariable and multivariable analyses utilized a Cox proportional hazards model to identify factors linked to recurrence. Statistical procedures were carried out using SPSS and R software, with a two-sided *P* < 0.05 deemed statistically significant.

## Results

### Baseline characteristics of enrolled patients

Ultimately, the MWA and surgery groups included 126 and 230 women and 32 and 58 men, respectively, with 0.65% (1/154) and 4.51% (13/288) located in the isthmus. The follow-up periods for the MWA and surgery groups were 18 months (range 12–30) and 18 months (range 12–24), respectively. Patients in the MWA group were younger than those in the surgery group (38 (range 30.75–47) vs 43 (range 34–50.75), *P* = 0.000). However, there was no significant difference in the proportion of patients under 55 years between the two groups. In the MWA group, both the maximum diameter (5.7 mm (range 4.6–7.0) vs 6.9 mm (range 5.8–8.6), *P* = 0.000) and the volume (70.7 cm^3^ (range 35.2–120.9) vs 122.0 cm^3^ (range 63.9–228.8), *P* = 0.000) were smaller than those in the surgery group. After 1:1 PSM, the baseline characteristics between the two groups were comparable, with 141 patients in each group (Table 1).

**Table 1:** Baseline characteristics of patients.

Characteristics	Before PSM			After PSM		
	MWA group (*n* = 158)	Surgery group (*n* = 288)	*P*	MWA group (*n* = 141)	Surgery group (*n* = 141)	*P*
Sex						
Female	126	230	0.977	112	105	0.323
Male	32	58		29	36	
Age, years	38 (30.8, 47)	43 (34, 50.8)	0.000	39 (31.5, 47.5)	42 (35.5, 49)	0.530
<55	144	247	0.099	127	130	
≥55	14	41		14	11	
Location						
Right/left	71/86	152/123		62/78	61/78	
Isthmus	1	13	0.065	1	1	1.00
Maximum tumor diameter, mm	5.7 (4.6, 7.0)	6.9 (5.8, 8.6)	0.000	5.9 (4.9, 7.2)	6.1 (5.2, 7.0)	0.303
Volume, mm^3^	70.7 (35.2, 120.9)	122.0 (63.9, 228.8)	0.000	78.4 (42.5, 126.8)	75.4 (51.1, 142.2)	0.326
Follow-up time, months	18 (12, 30)	18 (12, 24)	0.918	18 (12, 30)	18 (15, 24)	0.552

PSM, propensity score matching; MWA, microwave ablation.

### Primary outcomes in the MWA and surgery groups

During the monitoring period, six patients in the MWA group developed LTP. Of these, five underwent salvage surgery and one received AS. US identified suspicious nodules and/or LNM, leading to salvage surgery for patients 1 and 4 at the 1 year follow-up. Patient 1 underwent a total thyroidectomy (TT), and postoperative pathology confirmed PTMC. Patient 4 received a TL, and the postoperative pathology revealed new PTMC and LNM in the residual thyroid lobe. One year post-MWA, patient 2 exhibited malignant ultrasonic features in the isoechoic nodule initially located in the thyroid isthmus. FNAC confirmed its malignancy. This patient then accepted TL and was pathologically diagnosed with ipsilateral PTMC with LNM. Patient 3, despite normal US findings, underwent TL because of anxiety; pathology revealed no residual tumor but confirmed LNM. Patient 5, eight months post-MWA, underwent FNAC and molecular testing, confirming LNM. This patient chose AS for personal reasons. Patient 6, at seven months post-MWA, had FNAC on hypoechoic nodules in the isthmus, indicating potential malignancy. Subsequent salvage surgery and pathology identified new PTMC. Another patient underwent salvage surgery at nine months because of anxiety, with pathology showing no residual tumor or LNM. Overall, there were two cases each of local recurrence, LNM and combined local recurrence with LNM post-MWA. No distant metastases were detected (Table 2).

**Table 2:** Characteristics of patients who developed local tumor progression.

No.	Group	Sex	Age	LTP	RFS	Location	Treatment
1	MWA	Female	40	New PTMC	12	Left	TT
2	MWA	Male	29	Residual tumor + LNM	3	Right	TL
3	MWA	Female	33	LNM	12	Left	TL
4	MWA	Male	47	New PTMC + LNM	12	Left	TL
5	MWA	Female	20	LNM	8	Left	AS
6	MWA	Female	45	New PTMC	7	Left	TT
7	Surgery	Female	44	LNM	12	Region III	AS
8*	Surgery	Female	34	New PTMC	18	Left	AS
9	Surgery	Male	51	LNM	8	Region III	AS

* The patient was not included in the surgery group for further analysis after propensity score matching.

LTP, local tumor progression; MWA, microwave ablation; RFS, recurrence-free survival; PTMC, papillary thyroid microcarcinoma; LNM, lymph node metastasis; TT, total thyroidectomy; TL, thyroid lobectomy; AS, active surveillance.

During the follow-up period, three patients in the surgery group developed LTP. Patient 7 underwent TL because of PTMC in the right lobe, and 12 months later, US revealed abnormal echogenicity in the right cervical lymph nodes. Patient 8, previously diagnosed with PTMC in the isthmus, underwent an expanded isthmectomy. Regrettably, PTMC emerged in the left lobe 18 months postoperatively. Patient 9 presented with PTMC in the right lobe pre-surgery and underwent TL. Eight months post-surgery, US revealed abnormal echogenicity in the cervical lymph nodes, and one month later, a secondary suspicious lymph node focus was identified. All three patients are currently under AS. No instances of distant metastasis or cancer-related deaths occurred during the follow-up period in either the MWA or surgery groups (Table 2).

**Figure 2:**
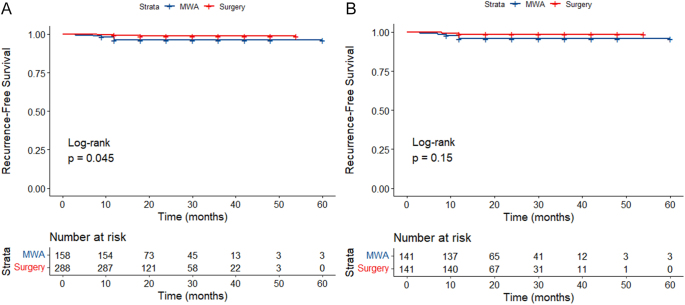
Comparison of RFS rates between the MWA and surgery groups (A: before PSM; B: after PSM).

Before PSM, the total incidence rates of LTP, LNM, new PTMC and tumor residue in the MWA group were 3.80, 2.53, 1.95 and 0.65%, respectively, compared with 1.04, 0.59, 0.35 and 0.005% in the surgery group. Before PSM, a significant difference in LTP rates was observed between the groups. However, post-PSM, no significant difference in LTP rates was noted (Table 3). Prior to PSM, RFS rates at 1 and 4 years were 99.31 and 98.82% in the surgery group and 96.19 and 96.19% in the MWA group (*P* = 0.045). Following PSM, the RFS rates at 1 and 4 years were 98.58 and 98.58% in the surgery group compared with 95.73 and 95.73% in the MWA group (*P* = 0.15) (Fig. 2). Cox regression analysis indicated that the treatment modality was not significantly associated with recurrence after adjusting for age, sex, tumor location and size (HR = 3.75, 95% CI: 0.94–14.98, *P* = 0.062) (Table 4).

**Table 3:** The comparison of local tumor progression between the two groups.

Variable	Before PSM			After PSM		
	MWA group (*n* = 158)	Surgery group (*n* = 288)	*P*	MWA group (*n* = 141)	Surgery group (*n* = 141)	*P*
LTP	6	3	0.045	6	2	0.152
LNM	4	2		4	2	
New PTMC	3	1		3	0	
Residual tumor	1	0		1	0	

MWA, microwave ablation; LTP, local tumor progression; LNM, lymph node metastasis; PTMC, papillary thyroid microcarcinoma; PSM, propensity score matching.

**Table 4:** Univariate and multivariate analysis evaluating the risk factors for recurrence.

Variable	Before PSM				After PSM			
	Univariate analysis		Multivariate analysis		Univariate analysis		Multivariate analysis	
	HR (CI)	*P*	HR (CI)	*P*	HR (CI)	*P*	HR (CI)	*P*
Age, year								
<55	0.04 (0.00, 246.98)	0.471	5.78 × 10^5^ (0.00, not reached)	0.984	0.04 (0.00, 1862.48)	0.564	5.78 × 10^5^ (0.00, not reached)	0.986
≥55								
Sex								
Male	1.96 (0.49, 7.86)	0.340	2.31 (0.56, 9.45)	0.244	2.03 (0.49, 8.51)	0.331	2.42 (0.57, 10.18)	0.230
Female								
Location								
Right/Left	0.26 (0.03, 2.05)	0.199	0.13 (0.01, 1.19)	0.070	20.30 (0.00, 1.84 × 10^17^)	0.872	5.31 × 10^5^ (0.00, not reached)	0.996
Isthmus								
Maximum tumor diameter, mm								
<5.0	0.41 (0.11, 1.65)	0.212	1.31 (0.29, 5.81)	0.726	0.43 (0.10, 1.80)	0.249	1.99 (0.47, 8.46)	0.351
≥5.0								
Treatment								
MWA	3.75 (0.94, 14.98)	0.062	4.07 (0.86, 19.18)	0.076	3.04 (0.61, 15.08)	0.173	3.11 (0.61, 15.81)	0.170
Surgery								

PTMC, papillary thyroid microcarcinoma; HR, hazard ratio; PSM, propensity score matching; MWA, microwave ablation.

**Figure 3:**
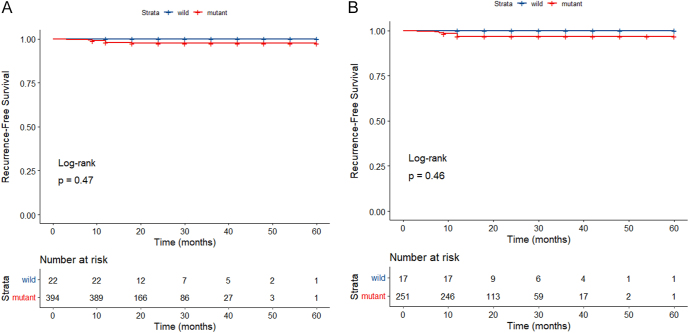
RFS rates for mutant and wild-type BRAFV600E PTMC patients. (A: before PSM; B: after PSM).

**Figure 4:**
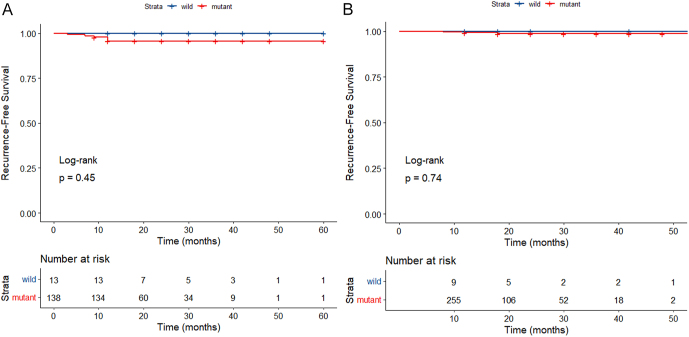
RFS rates for mutant and wild-type BRAFV600E PTMC patients (A: MWA group; B: surgery group).

Mutant BRAFV600E was identified in CNB and/or FNAC in 138 patients in the MWA group and 256 patients in the surgery group prior to PSM, while wild-type BRAFV600E was found in 13 and 9 patients, respectively. Following PSM, mutant BRAFV600E was detected in 121 patients in the MWA group and 130 in the surgery group, with the wild type identified in 13 and 4 patients, respectively. No significant differences were observed in RFS between patients with mutant and wild-type BRAFV600E PTMC both before and after PSM (*P* = 0.47 and 0.46) (Fig. 3). Similarly, no significant differences in RFS were noted between patients with mutant and wild-type BRAFV600E PTMC in the MWA and surgery groups (*P* = 0.45 and 0.74) (Fig. 4).

### Secondary outcomes of patients in MWA and surgery groups

In the surgery group, complications included two cases of postoperative laryngeal discomfort, one patient with ecchymosis around the wound, one patient with fresh bleeding post-operation who was transferred to the ICU, another patient moved to the ICU because of a postoperative infection, one patient who experienced neck pain for over two years and one patient with occasional numbness in the right hand.

In the MWA group, the overall complication rate was 2.53%, with major complications at 0.63% and minor complications at 1.90%. In the surgery group, the overall complication rate was also 2.43%, with major complications at 0.005%, and the rate of minor complications was equal to the overall rate at 2.43%. The difference in complication rates between the two groups was not statistically significant (Table 5).

**Table 5:** The comparison of complications of the two groups.

Complications	MWA group	Surgery group	*P*
Overall complications	4	7	1.00
Major complications			0.354
Recurrent laryngeal nerve injury	1	0	
Hypoparathyroidism	0	0	
Minor complications	3	7	0.977

MWA, microwave ablation.

Patients in the surgery group experienced significantly longer procedure times (90 (75, 115) vs 1.17 (0.83, 1.77) minutes, *P* < 0.001), greater blood loss (60 (45, 90) vs 0 mL, *P* < 0.001), longer hospital stays (7 (6, 8) vs 4 (3, 4) days, *P* < 0.001) and higher costs (23,232.57 (20,663.54, 25,981.17) vs 16,973.9 (15,794.1, 18,173.65) Yuan, *P* < 0.001) than those in the MWA group (Table 6).

**Table 6:** The comparison of treatment variables of the two groups.

Variable	MWA group	Surgery group	*P*
Hospitalization, day	4 (3, 4)	7 (6, 8)	<0.001
Procedure time, minute	1.17 (0.83, 1.77)	90 (75, 115)	<0.001
Estimated blood loss, mL	0	60 (45, 90)	<0.001
Cost, yuan	16,973.9 (15,794.1, 18,173.65)	23,232.57 (20,663.54, 25,981.17)	<0.001

MWA, microwave ablation.

### Other outcomes

In total, 246 patients opted for TL, 25 for TT, 13 for subtotal thyroidectomy and four for extended isthmus resection. Among those who chose TL, three underwent a neck approach, 33 underwent an oral vestibular approach, five underwent a breast approach, one underwent an axillary approach and the remainder underwent open surgery.

Surgical pathology indicated that the incidence of occult CLNM and PTMC was 28.47% (82/288) and 7.64% (22/288), respectively. Among patients with occult lesions, three were not diagnosed with thyroid malignancy by FNAC on lateral lobe nodules before surgery, but subsequent pathology revealed occult PTMC. Additionally, two patients exhibited occult PTMC on the lateral lobe and one had a cystic-solid nodule on the contralateral lobe, which was confirmed as PTMC post-surgery. PTMC was also found in one patient with a thyroglossal duct cyst. Occult PTMC was identified in the ipsilateral lobe in 13 patients postoperatively. Two patients underwent extended isthmus resection, with pathology subsequently revealing occult PTMC in the thyroid lobe.

## Discussion

Previous studies have demonstrated that TA and surgical treatment of low-risk PTMC offer comparable efficacy and safety, although long-term efficacy studies remain sparse and localized to a few centers. The invasiveness of mutant BRAFV600E PTMC and the effectiveness and safety of MWA in treating this variant require further investigation. In this study, PSM was utilized to compare the efficacy and safety of MWA and surgery in treating low-risk PTMC. The results indicated that MWA had significantly lower procedure times, hospital stays and costs compared to surgery, with no significant differences in the incidence of LTP, LNM or complication rates. These findings suggest that MWA is an effective and safe option for treating low-risk PTMC. After adjusting for age, sex, tumor location and maximum diameter, treatment modality did not significantly correlate with LTP. Additionally, mutant BRAFV600E was not significantly associated with LTP.

Earlier research has validated the efficacy and safety of TA. Yan *et al.* (8) analyzed 884 patients with unifocal low-risk PTMC treated with TL or RFA. After 1:1 PSM, each group comprised 332 patients. Over a follow-up period of 48.3 months, there were no significant differences between the groups in terms of LTP (1.8 vs 3.3%, *P* = 0.209), LNM (0.6% vs 0.6, *P* = 1.000), recurrent PTMC (1.2 vs 2.4%, *P* = 0.244), persistent lesions (0 vs 0.3%, *P* = 0.317) and 4-year RFS, indicating that RFA is a viable surgical alternative. While some studies have affirmed the safety and efficacy of MWA for treating low-risk PTMC (12), comparisons of long-term outcomes between MWA and surgical interventions are limited. This study proposes that MWA may be a viable alternative treatment for low-risk PTMC.

However, it is important to note that low-risk PTMC may harbor occult lesions and CLNM. The meta-analysis revealed a 26.6% prevalence of contralateral occult PTC in patients undergoing TT for unilateral PTC (13). Among clinically solitary PTMC patients, 3.7% had ipsilateral occult carcinoma, while 15.5–22.3% had contralateral occult carcinoma (14, 15). This study found that the incidence of occult lesions was 7.64% (22/288), with ipsilateral and contralateral occult lesions at 2.08% (6/288) and 5.21% (15/288), respectively. The detection rate of contralateral occult lesions may be under-represented as the patients in the surgery group were selected based on criteria suitable for TA, predominantly underwent TL and those with suspected multifocal lesions were excluded preoperatively. Another meta-analysis indicated a CLNM incidence of 33% (95% CI: 29–37) in cN0 PTMC patients (16). The incidence of CLNM in this study was 28.47% (82/288), aligning with prior findings. Jin *et al.* (17) suggested that more than a third of low-risk PTMC patients, deemed suitable for TA, might possess occult lesions. Thus, a thorough pre-TA assessment for occult lesions is essential, particularly in patients with high-risk factors, despite numerous studies indicating that occult lesions may minimally impact overall survival rates (3).

Compared with surgery, MWA offers several advantages including reduced costs, shorter procedure times and decreased hospital stays, which are beneficial for patient recovery. Previous studies have indicated a lower incidence of complications with TA compared with surgery (8). However, in this study, the complication rates between the two groups were similar, possibly because of the expertise of the surgeons involved. Additionally, one patient in the MWA group underwent salvage surgery due to anxiety, in contrast to 32–69% of patients who underwent salvage surgery for the same reason during AS (18). This suggests that TA might reduce or even eliminate patient anxiety by inactivating primary tumors, although further verification through a prospective controlled study is needed.

Some research suggests that the mutant BRAFV600E variant in PTMC is associated with increased thyroid cancer invasiveness. Previous findings suggest that mutant BRAFV600E intrathyroidal PTC >1.0 cm has a substantially increased recurrence risk, particularly in the case of tumors >2.0 cm where there was a robustly increased recurrence risk to around 20–30%, which was comparable with the recurrence risk of invasive PTC (19). Oh *et al.* (20) included 743 patients treated with TT for PTMC and found that recurrences were 1.3 versus 4.3% in wild-type BRAF versus mutant BRAF patients, with an HR of 6.65 (95% CI, 1.80–24.65) after adjustment for confounding clinical factors on the analyses of low-risk PTMC. The BRAF mutation is linked to a significant reduction in RFS in low-risk PTMC even though the recurrence rate is relatively low. Similar findings have been found in a study conducted by Kim *et al.* (21). A meta-analysis further confirmed that mutant BRAFV600E PTMC is more invasive than its wild-type counterpart (22). However, some studies hold different opinions. Won *et al.* (23) retrospectively reviewed 2094 PTMC patients who underwent surgery and had a valid BRAFV600E mutation test result. The results suggested that the BRAFV600E mutation could not accurately predict LNM or the recurrence of Chinese PTMC patients. In addition, there was no correlation between the BRAF mutation status and recurrence rate in low-risk PTMC patients who underwent AS. Ramone *et al.* found that the comparison between the molecular profile and the clinical outcome of the PTMC (stable versus progressive disease) showed no correlation and did not identify a molecular signature such as BRAF, RAS or gene fusions, able to identify progressive PTMC (24). As for TA, Lin *et al.* (9) retrospectively analyzed 60 mutant BRAFV600E PTMC patients, finding no instances of LTP or CLNM, with voice change (1.7%) being the only major complication. Consequently, some researchers consider RFA to be an effective and safe treatment for single-focus mutant BRAFV600E PTMC. In this study, the results showed that recurrence was not associated with the BRAFV600E mutation status. Thus, the influence of BRAFV600E on long-term prognosis of PTMC after treatment seems to be limited, and the BRAFV600E mutation is unlikely a contraindication for TA.

At present, TA is considered as one of the treatments for low-risk PTMC. AS or nonsurgical treatments (such as TA) are not appropriate for patients with tumors with high-risk features, such as clinical metastasis at diagnosis, signs or symptoms of invasion to the RLN or trachea and high-grade malignancy on cytology or for those with tumors involving critical anatomic fields (near the trachea or located in the pathway of the RLN) (25). TA is suitable for low-risk PTMC, similar to those patients who are candidates for AS but would like to actively treat their cancer (26). Some studies have shown that TA is effective and safe for T1bN0M0 PTC (27). Moreover, PTMCs in the isthmus (28), multifocal PTMCs (29) and PTMCs adjacent to the dorsal capsule (30) may not be contraindications for ablation. An expanding body of research has been dedicated to broadening the applicability of TA, initially from recurrent thyroid cancer and lymph nodes to now encompass isolated PTMC alongside a comprehensive exploration into the expanded parameters such as the size, number and location of PTMC and its applicability in other types of thyroid cancer (31). It is worth noting that the efficacy and safety of TA should not arouse the interest in early diagnosis and overtreatment of low-risk PTMC. In addition, more large-sample data from various regions around the world and prospective trial designs are required to establish reliable evidence regarding the long-term efficacy of TA.

This study has some limitations. First, this is a retrospective study. While randomized clinical trials provide the most definitive conclusions, they are challenging to implement clinically because of patient preferences. Thus, in the absence of prospective or randomized clinical trials, PSM analysis suggests that the outcomes of MWA and surgical treatment for low-risk PTMC are comparable, providing a robust level of evidence-based support. Second, the follow-up period was brief. Given that PTMC patients generally have a favorable prognosis, a longer follow-up is necessary to validate the findings. Third, the surgical group underwent various surgical techniques, which could influence postoperative pathology outcomes, although existing studies indicate that different surgical methods yield similar efficacy and safety (32). In the future, prospectively multicentered studies with a large sample should be designed and conducted to compare the long-term efficacy and safety of AS, TA and surgery on low-risk PTMC patients. The quality of life and psychological state of patients are required to be further evaluated and compared.

## Conclusions

The clinical efficacy and safety of MWA and surgical treatment for low-risk PTMC are equivalent. As a minimally invasive option, MWA may represent a safe and effective alternative for the surgical management of low-risk PTMC patients. MWA is also a promising alternative for treating low-risk PTMC with the BRAFV600E mutation.

## Declaration of interest

The authors declare that they have no competing interest. Shuang Xu is a Senior Editor of Endocrine Connections. Shuhang Xu was not involved in the review or editorial process for this paper, on which he is listed as an author.

## Funding

This study was funded by the Jiangsu Provincial Key Research and Development Program (Grant No. BE2020726), the Central fiscal transfer payment local project – traditional Chinese medicine evidence-based capacity promotion project (Grant No. 2023ZYCZ-001) and the Open project of the National Traditional Chinese Medicine Clinical Research Base (Grant Nos. JD2022SZXZD05 and JD2023SZX08).

## Author contribution statement

Yujie Ren and Shuhang Xu developed the research questionnaire and drafted the protocol for this study. Yujie Ren and Yujiang Li were responsible for data collection and analysis. Xiaoqiu Chu, Chao Liu and Guofang Chen participated in the diagnosis. Shuhang Xu performed MWA. Jianhua Wang performed thyroid surgery. Xue Han and Yueting Zhao were responsible for the perioperative management. Yujie Ren drafted the manuscript. Shuhang Xu and Chao Liu revised the manuscript critically for important intellectual content. All authors agreed to take responsibility for the integrity of the data and the accuracy of data analysis and approved the final version of the manuscript.

## Data availability

The datasets generated and analyzed in the present study are available from the corresponding author upon reasonable request.
